# Advanced Parkinson’s disease treatment patterns in Italy: an observational study interim analysis

**DOI:** 10.1080/07853890.2024.2315226

**Published:** 2024-02-21

**Authors:** Fabrizio Stocchi, Paolo Barone, Roberto Ceravolo, Maria Francesca De Pandis, Leonardo Lopiano, Nicola Modugno, Alessandro Padovani, Manuela Pilleri, Alessandro Tessitore, Mario Zappia

**Affiliations:** a Department of Neurology, University San Raffaele Roma and IRCCS San Raffaele, Rome, Italy; b Centro per le Malattie Neurodegenerative, Dipartimento di Medicina, Università di Salerno, Baronissi, Salerno, Italy; c Neurodegenerative Disease Center, Department of Clinical and Experimental Medicine, University of Pisa, Pisa, Italy; d Department of Human Sciences and Promotion of Quality of Life, San Raffaele University, Roma, Italy; e San Raffaele Cassino Hospital, Cassino, Italy; f Department of Neuroscience Rita Levi-Montalcini, University of Turin AOU Città della Salute e della Scienza, Turin, Italy; g I.R.C.C.S. Neuromed, Pozzilli, Isernia, Italy; h Unità di Neurologia, Dipartimento Scienze Cliniche e Sperimentali, Università degli Studi di Brescia, Brescia, Italy; i UO Neurologia Casa di Cura Villa Margherita, Arcugnano Vicenza, Italy and Centro Parkinson e Parkinsonismi, ASST Gaetano Pini CTO, Milano, Italy; j Department of Advanced Medical and Surgical Sciences, University of Campania "L. Vanvitelli", Naples, Italy; k Department “G.F. Ingrassia”, University of Catania, Catania, Italy

**Keywords:** COMT inhibitor, levodopa, MAO-B inhibitor, motor/non-motor fluctuations, parkinson disease

## Abstract

**Background:**

Oral levodopa remains the mainstay of treatment for Parkinson’s disease (PD). However, as PD progresses, response to treatment may fluctuate. Managing fluctuations can be demanding for clinicians and patients. There is a paucity of real-world studies reporting on PD management in patients with fluctuations in treatment response, especially in patients with advanced stages of PD. The multicentre, observational Parkinson’s Disease Fluctuations treatment PAthway (PD-FPA) study describes the real-life management of response fluctuations in Italian patients with advanced PD.

**Patients and Methods:**

PD-FPA had a retrospective and prospective phase; herein, retrospective results are presented. Ten Italian centres enrolled patients with a PD diagnosis from 10–15 years prior to study entry (T0) and who had ≥2-year history of fluctuations. Data on patient demographics, medical history, PD stage, fluctuation characteristics, symptoms, and prescribed treatments were collected at T0 and retrospectively (2 years prior to T0) *via* patient chart review/interview.

**Results:**

Overall, 296 patients (60% male, mean age 68 years, 84% with Hoehn and Yahr scores 2–3) were enrolled. At T0, most patients (99.3%) were on oral levodopa therapy. All patients used dopaminergic medications; adjunctive medications included dopamine agonists (56%) and monoamine oxidase B (60%) and catechol-O-methyltransferase enzyme inhibitors (41%). At T0, 51% of patients had changed therapy, with response fluctuations being the most common reason (74%); wearing-off was the most common fluctuation (83%).

**Conclusion:**

This interim analysis of PD-FPA suggests that adequate levodopa dosing and adjunctive medications can stabilize advanced PD and provide patients with a good quality of life.

## KEY MESSAGES

Patients with Parkinson’s disease (PD) often exhibit fluctuations in their response to oral levodopa; however, real-world studies on the management of these fluctuations are lacking. This planned interim analysis of the real-world, multicentre, observational PD Fluctuations treatment Pathway (PD-FPA) study found that adequate levodopa dosing and adjunctive medications can stabilize Italian patients with advanced PD and improve their quality of life.

## 1. Introduction

Parkinson’s disease (PD), the second most common neurodegenerative disorder after Alzheimer’s disease, is caused by the gradual loss of dopaminergic neurons in the substantia nigra pars compacta [1]. PD is characterized by motor symptoms, including tremors, rigidity, bradykinesia/akinesia, and postural instability [1]. Non-motor symptoms include cognitive impairment, depression, anxiety, psychosis, autonomic and gastrointestinal dysfunction, and pain and sensory alterations [1].

Levodopa, an orally administered dopamine precursor, has been the mainstay of symptomatic PD treatment for several decades. Although initial levodopa treatment provides substantial benefits, chronic levodopa use (i.e. 2–5 years) has been associated with complications, fluctuations in treatment response, and rapid re-emergence of motor/non-motor symptoms and dyskinesias [2–5]. Disease progression and the development of complications due to the pharmacokinetics of levodopa [3,6] considerably affect patients’ daily activities and quality of life [7,8].

Fluctuations in treatment response are usually managed by increasing the levodopa dose or frequency of administration or by initiating oral adjunctive medications, such as dopaminergic agonists or catechol-O-methyltransferase (COMT) and monoamine oxidase B (MAO-B) enzyme inhibitors [2,9,10]. As PD progresses, invasive or device-aided treatments, including deep brain stimulation (DBS), continuous subcutaneous delivery of medication (apomorphine pump), or levodopa-carbidopa intestinal gel (LCIG) infusions, are recommended [6,11,12].

Despite the availability of several treatment options beyond standard dopamine replacement therapy, the management of patients with advanced PD is challenging. Clinicians and patients must decide when to introduce additional drugs to regimens, when to modify the dosage of levodopa, and how to select the most appropriate advanced therapy [12].

Currently, there is a paucity of studies reporting on PD management in patients with fluctuations in treatment response in the real world [13].

The Parkinson’s Disease Fluctuations treatment PAthway (PD-FPA) study was an Italian, multicentre, observational study designed to provide insights into the real-life management of motor fluctuations experienced by patients with advanced PD in Italy. Herein, we report the results of a planned interim analysis of the PD-FPA study.

## 2. Patients and methods

### 2.1. Study design and patients

**Figure 1.**
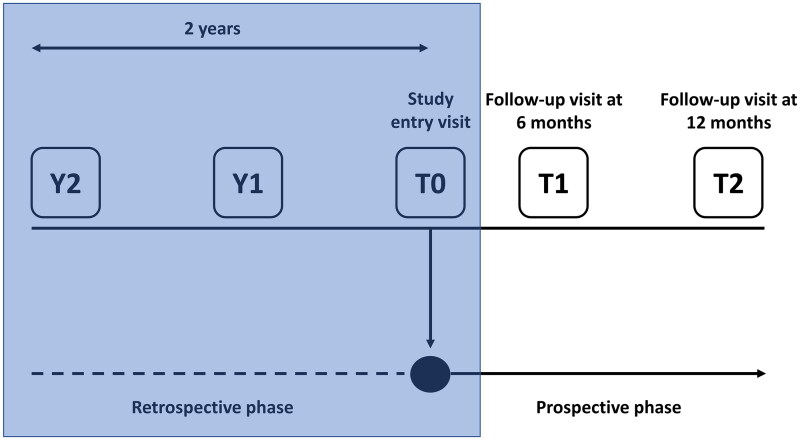
Study design (the phase discussed in this manuscript is in the shaded box). T0, study entry; Y, year prior to study entry.

The PD-FPA study was a multicentre, observational study with a retrospective and prospective phase (Figure 1). This article reports the results of a planned interim analysis focused on the retrospective phase; results from the prospective phase will be reported separately.

Ten hospitals, recognized as centres of reference for PD, from across Italy (four hospitals in southern Italy, two in central Italy, and four in northern Italy) participated in the study. Each centre consecutively enrolled patients aged ≥18 years with a PD diagnosis from 10–15 years before enrolment and who had been experiencing fluctuations in treatment response with oral levodopa for ≥2 years. Patients who had undergone neurosurgery (i.e. DBS) were not eligible for enrolment, and those who withdrew consent or were lost to follow-up were excluded from the final analysis. The study was approved by the ethics committee of IRCCS San Raffaele Pisana, Rome, Italy (05/2018), and all patients provided written informed consent upon enrolment.

### 2.2. Study objectives

The primary objective of the PD-FPA study was to describe how motor fluctuations in PD are treated in routine clinical practice in Italy. Secondary objectives were to characterize patients with advanced PD and motor fluctuations; analyse relationships and associations between motor fluctuations and patient characteristics or PD treatments, respectively; describe PD motor/non-motor symptoms, their incidence and their association with patient characteristics and therapy; and evaluate how frequently infusion therapy and neurosurgery are utilized. The retrospective phase of PD-FPA focused on describing patients with long-standing PD and fluctuations in treatment response, and providing an overview of treatments used in clinical practice. All secondary objectives were completed in the prospective phase and are not discussed in this analysis.

### 2.3. Variables analysed

Information on patient demographics, medical history, PD stage, fluctuation characteristics, motor and non-motor symptoms, PD treatment and comorbidities were collected *via* patient interviews during a centre visit at study entry (T0) and retrospectively from patient charts at timepoints corresponding to 1 (Y1) and 2 (Y2) years prior to T0 (Figure 1). In the prospective phase, patients were followed up for 1 year and seen at two visits, approximately 6 (T1) and 12 months (T2) after the initial visit (Figure 1). All data were recorded using study case report forms.

### 2.4. Assessments

Clinical assessments were performed at T0, T1 and T2 (Figure 1). PD staging was determined by the Hoehn and Yahr scale [14]. Fluctuations in treatment response were assessed according to the Italian version of the self-administered 19-item Wearing-Off Questionnaire (WOQ-19) [15,16], which screens for the presence of 19 motor/non-motor PD symptoms and whether they have improved with medication. Motor/non-motor PD symptoms were assessed using the Unified Parkinson’s Disease Rating Scale by the Movement Disorder Society (MDS-UPDRS) [17], the Non-Motor Symptom Scale (NMSS) [18,19], and the Italian version of the 39-item, self-administered Parkinson’s Disease Questionnaire (PDQ-39) [20–22]. The MDS-UPDRS consists of four major parts: part I (non-motor experiences of daily living), part II (motor experiences of daily living), part III (motor examination), and part IV (motor complications) [17]. Part I is further subdivided into two components: IA, in which the physician assesses several behaviours with relevant information from patients and caregivers, and IB, which is completed by the patient. Part II is also self-administered. The NMSS is a 30-item questionnaire encompassing nine relevant domains: cardiovascular, sleep/fatigue, mood/cognition, perceptual problems, attention/memory, gastrointestinal, urinary, sexual function, and miscellaneous aspects [18]. PDQ-39 is self-administered, assesses PD impact on functioning and well-being, and contains 39 items encompassing eight domains: mobility (10 items), activities of daily living (6 items), emotional well-being (6 items), stigma (4 items), social support (3 items), cognitions (4 items), communication (3 items), and bodily discomfort (3 items) [20]. Each domain is scored by expressing the sum of the item scores as a percentage ranging from 0 to 100, where 100 indicates maximum impact.

### 2.5. Statistical analysis

This interim analysis includes data collected at T0 and retrospective data from Y1 and Y2. Data from these time points were summarized using descriptive statistics. Frequency and percentages were reported for categorical variables, and mean and standard deviation (SD) values were reported for continuous variables. The analyses were conducted using SAS software version 9.4 (SAS Institute Inc., Cary, NC, USA).

## 3. Results

### 3.1. Patient characteristics

Overall, 296 patients with advanced PD were consecutively enrolled from 10 centres (114/296 [38.5%] patients were from southern Italy, 74 [25.0%] from central Italy, and 108 [36.5%] from northern Italy). Demographic and clinical characteristics reported by the patients at T0 are summarized in Table 1.

**Table 1.** Patient demographic and clinical characteristics.

Characteristics	Patients (*N* = 296)	
	n (%)	Mean (± SD)
Sex		
Male	178 (60.1)	–
Female	118 (39.9)	–
Age, years		
At T0	–	68.0 (9.7)
At diagnosis	–	55.3 (9.8)
At first fluctuation	–	62.6 (10.6)
Disease duration, years	–	12.6 (4.4)
Oral levodopa dose		
Yes	294 (99.3)	–
Total daily dose, mg	–	662.2 (271.5)
Number of daily administrations	–	5.6 (1.9)
Time from last administration, min	130	–
LCIG use	13 (4.4)	–
Hoehn and Yahr Stage^a^		
0	1 (0.3)	–
1	8 (2.7)	–
2	127 (42.9)	–
3	120 (40.5)	–
4	33 (11.1)	–
5	6 (2.0)	–
Motor examination score^b^ MDS-UPDRS, Part III		
All	–	37.6 (16.2)
NMSS	–	59.1 (43.2)
PDQ-39	–	35.0 (14.7)
Presence of comorbidities	139 (47.0)	–
Comorbidities affecting ≥5% of patients^c^		
Hypertension	63 (21.3)	–
Anxiety/Depression	29 (9.8)	–
Heart disease^d^	25 (8.5)	–
Benign prostatic hyperplasia	20 (6.8)	–
Dyslipidemia/Hypercholesterolemia	19 (6.5)	–
Gastritis/Gastroesophageal reflux	19 (6.5)	–
Diabetes	18 (6.0)	–
Osteoporosis	15 (5.0)	–

LCIG: levodopa-carbidopa intestinal gel; MDS-UPDRS: Unified Parkinson’s Disease Rating Scale by the Movement Disorder Society; NMSS: Non-Motor Symptom Scale; PD: Parkinson’s disease; PDQ-39: 39-item Parkinson’s Disease Questionnaire; SD: standard deviation; T0: study entry; –: not available.

^a^ PD stages according to the Hoehn and Yahr Scale: 0, Asymptomatic; 1, Unilateral involvement only; 2, Bilateral involvement without impairment of balance; 3, Mild-to-moderate involvement and some postural instability but physically independent. Needs assistance to recover from pull test; 4, Severe disability, still able to walk or stand unassisted; 5, Wheelchair bound or bedridden unless aided [16].

^b^ Score according to MDS-UPDRS, Part III (range 0–108).

^c^ Based on patient recall of comorbidities.

^d^ Angina, arrythmia, hypertensive heart disease, ischemic heart disease, atrial fibrillation, heart failure and valvular heart disease.

The mean age of the study population was 68.0 years, and 60.0% of patients were male. Mean disease duration at baseline was 12.6 years and nearly all patients (*n* = 294; 99.3%) were receiving oral levodopa, at a mean daily dose of 662.2 mg with a mean of 5.6 daily administrations; 4.4% of patients (*n* = 13) were receiving LCIG. According to the Hoehn and Yahr Scale, 43.0% of patients (*n* = 127) had stage 2 PD, 40.5% (*n* = 120) had stage 3, and 11.0% (*n* = 33) had stage 4. The mean motor score (part III of the MDS-UPDRS) in the overall population was 37.6 (i.e. moderate severity) [23]. In the overall population, the mean NMSS score for non-motor symptoms was 59.1 (range 0–237), and the mean PDQ-39 score was 35.0.

Almost half the patients reported having comorbidities; hypertension was the most common (21.3%), followed by anxiety/depression (9.8%) and heart disease (8.5%). The pattern of comorbidities at T0 was similar to that observed in the retrospective analyses of Y1 and Y2 data (Supplementary Figure S1), although a slight numerical increase in comorbidity frequency was observed over time.

### 3.2. PD features at study entry

**Figure 2.**
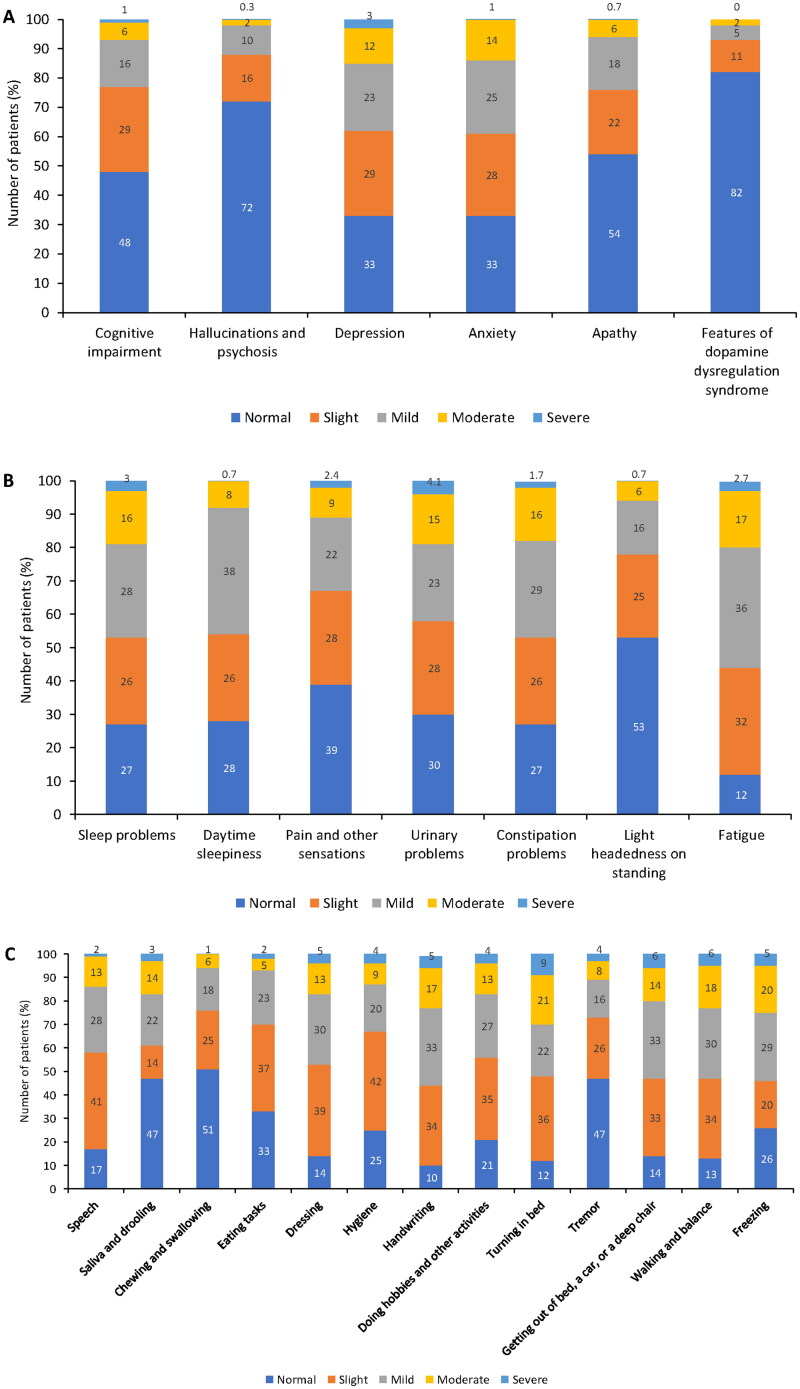
Non-motor and motor symptoms of Parkinson’s disease (assessed by parts I/II of MDS-UPDRS). (A) part IA (physician assessed) nM-EDL; (B) part IB (patient assessed) nM-EDL; and (C) part II (patient assessed) motor experiences of daily living. MDS-UPDRS: Unified Parkinson’s Disease Rating Scale by the Movement Disorder Society; nM-EDL: non-motor experiences of daily living.

Motor/non-motor PD symptoms were evaluated at T0 using the MDS-UPDRS; Figure 2 summarizes the results of part I and II of the questionnaire (i.e. motor/non-motor symptoms experienced in daily living). According to physician assessment (part I), anxiety and depression were the most prevalent non-motor symptoms; 40.0% of patients were considered to have anxiety and 36.4% to have depression (i.e. had symptoms of these disorders that were rated by physicians as ‘mild’, ‘moderate’ or ‘severe’; Figure 2(A)). Fatigue, sleep problems and daytime sleepiness were the non-motor symptoms most frequently reported by patients (55.7%, 47.0% and 46.7% of patients, respectively; Figure 2(B)). According to patient assessment (part II), handwriting (55.0%), walking and balance (54.0%), freezing (54.0%) and getting out of bed, a car, or a deep chair (53.0%) were the most commonly affected motor symptoms (Figure 2(C)).

Motor complications (dyskinesias, motor fluctuation, and OFF-state dystonia) were evaluated in part IV of the MDS-UPDRS; see Supplementary Table S1 for the full data set, including a subanalysis by sex. Dyskinesia impacted daily activities and social interaction of 59.5% of patients, among which the impact was slight-to-mild in 47.0% and moderate-to-severe in 12.0%. On average, patients experienced dyskinesia for 23.0% of their total waking hours. Dyskinesias were more frequently observed in females than in males (70.0% vs 53.0%). Overall, 44.0% of males and 53.0% of females reported slight-to-mild dyskinesia, and 9.0% and 17.0%, respectively, reported moderate-to-severe dyskinesia. On average, females reported dyskinesia for 26.0% of their total waking hours and males reported dyskinesia for 21.0% of their total waking hours.

Most patients (*n* = 269; 91.0%) spent ≤50% of their waking hours in the OFF state; on average patients spent 25.0% of their waking hours in the OFF state. Motor fluctuations had no impact in 7.5% of patients, a slight-to-mild functional impact in 58.0%, and a moderate-to-severe impact in 34.0%. OFF times were predictable most of the time in 81.0% of patients. No differences were observed between males and females in time spent in the OFF-state or the functional impact of fluctuations (Supplementary Table S1).

### 3.3. Response fluctuations over a 2-year period

**Figure 3.**
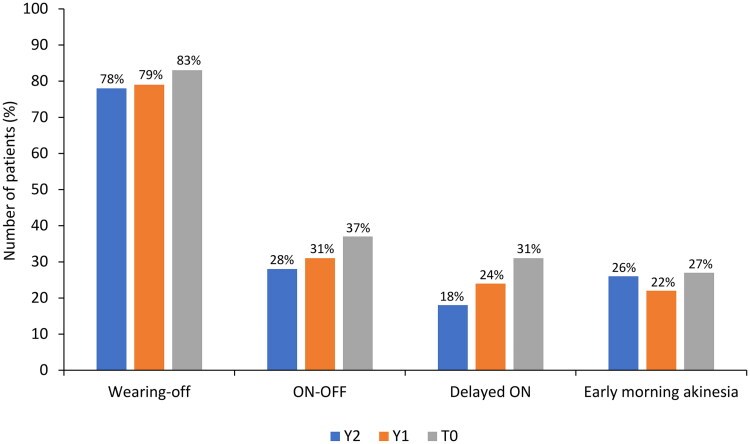
Types of motor fluctuations in the 2 years (Y2 and Y1) prior to study entry (T0).

**Figure 4.**
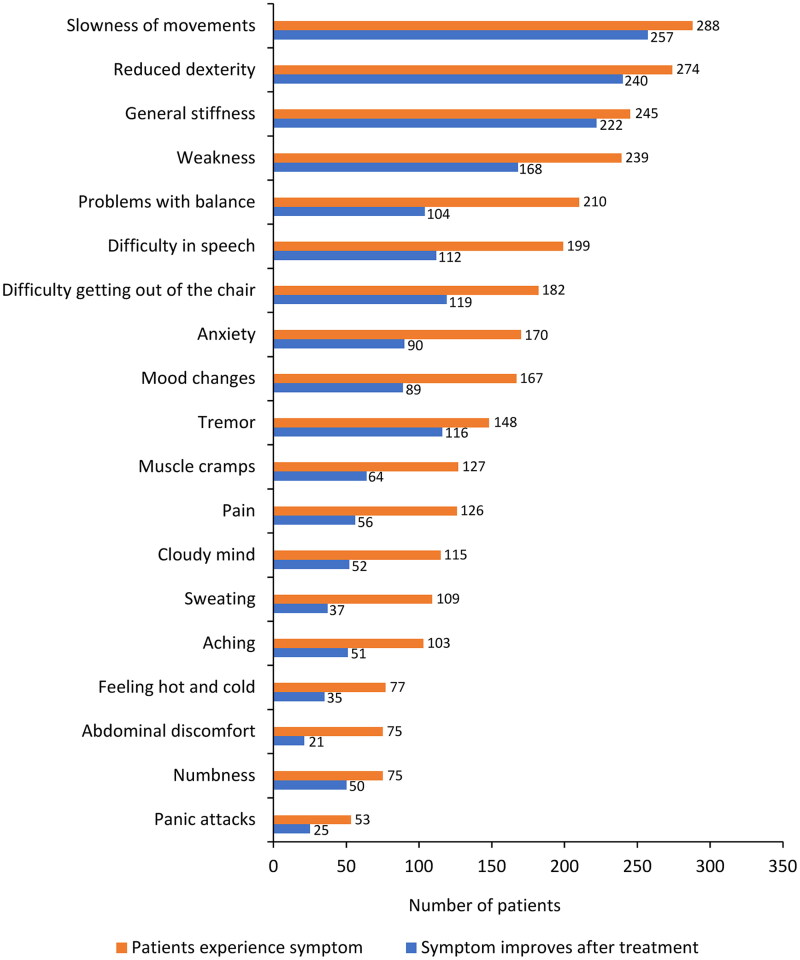
Improvements in motor and non-motor symptoms of wearing-off fluctuations (as assessed by the WOQ-19). WOQ-19: 19-item Wearing-Off Questionnaire.

The types of fluctuations in treatment response were evaluated at T0 and retrospectively at Y1 and Y2. At T0, wearing-off was the most common response fluctuation (*n* = 245/296, 83.0%), followed by the ON-OFF phenomenon (*n* = 109, 37.0%), delayed ON (*n* = 91, 31.0%), and early morning akinesia (*n* = 79, 27.0%; Figure 3). Over the 2 years before study entry, fluctuations in treatment response were similar to those at T0, although a numerical increase in fluctuation frequency was observed. Wearing-off was further investigated using the WOQ-19 questionnaire. Figure 4 summarizes the most commonly reported symptoms at T0, which included slowness of movements (*n* = 288/296, 97.0%), reduced dexterity (*n* = 274, 93.0%), general stiffness (*n* = 245, 83.0%), weakness (*n* = 239, 81.0%), problems with balance (*n* = 210, 71.0%), difficulty with speech (*n* = 199, 67.0%), difficulty getting out of a chair (*n* = 182, 61.0%), anxiety (*n* = 170, 57.0%), mood changes (*n* = 167, 56.0%), and tremor (*n* = 148, 50.0%). In the majority of patients (65.0–89.0%), most symptoms improved after taking levodopa. Of note, problems with balance, difficulty in speech, anxiety, and mood changes improved in fewer patients (50.0–56.0%) after taking levodopa (Figure 4).

### 3.4. Patterns of treatment over a 2-year period

**Figure 5.**
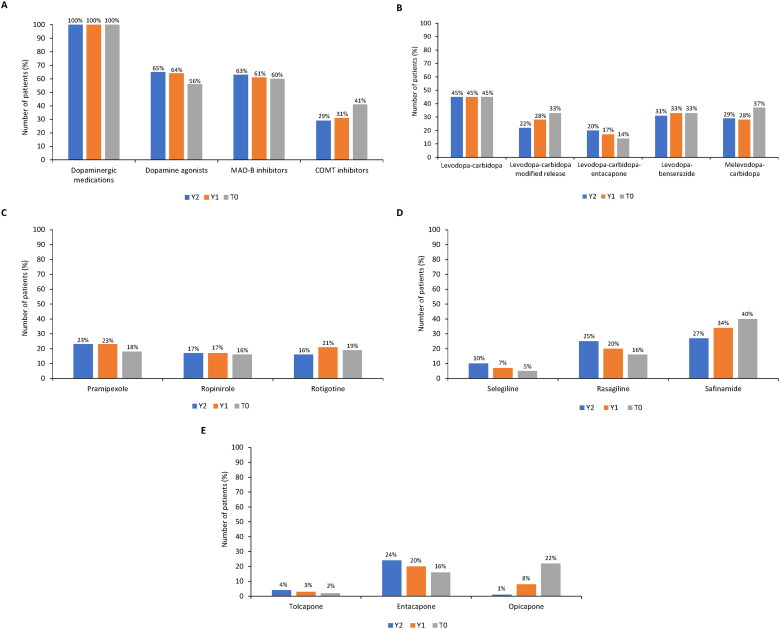
Parkinson’s disease treatments 2 years prior (Y2 and Y1) to study entry (T0). (A) pharmacologic treatment (levodopa-carbidopa-entacapone was included in two classes - dopaminergic medications and COMT inhibitors); (B) dopaminergic medications (including concomitant use of >1 medication or combination); (C) dopamine agonists; (D) MAO-B inhibitors; and (E) COMT inhibitors (entacapone included levodopa-carbidopa-entacapone). COMT: catechol-O-methyltransferase enzyme; MAO-B: monoamine oxidase B enzyme.

At T0, 99.3% (*n* = 294) of patients were receiving oral levodopa therapy and 4.4% (*n* = 13) were receiving infusion therapy. A slight numerical increase in infusion therapy was observed across the 2 years prior to study entry (Y2: 100.0% oral, 2.4% infusion; Y1: 99.9% oral, 3.4% infusion). At T0, all patients used dopaminergic medications; 56.0% (*n* = 165/296) of patients used dopamine agonists, 60.0% (*n* = 177) used MAO-B inhibitors, and 41.0% (*n* = 121) used COMT inhibitors (Figure 5(A)). A complete overview of all dopaminergic medications and dopamine agonists used is provided in Supplementary Tables S2 and S3.

Figure 5(B) summarizes the use of dopaminergic therapy. At T0, 78.0% of patients (*n* = 231/296) used levodopa + carbidopa (an aromatic L-amino acid decarboxylase [AADC] inhibitor) as either regular or modified-release tablets, 37.0% (*n* = 109) used melevodopa (levodopa methyl ester) + carbidopa, 33.0% (*n* = 97) used levodopa-benserazide (AADC inhibitor), and 14.0% (*n* = 41) used the triple combination levodopa-carbidopa-entacapone (COMT inhibitor). In the 2 years before study entry, the use of the modified-release formulation of levodopa-carbidopa numerically increased from Y2 to T0, as did the use of melevodopa-carbidopa. The use of levodopa-carbidopa-entacapone decreased, while levodopa-carbidopa and levodopa-benserazide remained stable.

Among dopamine agonists, rotigotine, pramipexole, and ropinirole were the most frequently prescribed, to 19.0%, 18.0%, and 16.0% of patients, respectively (Figure 5C). Use of these medications decreased slightly in the 2 years prior to T0. Safinamide was the most frequently prescribed MAO-B inhibitor (40.0% of patients), followed by rasagiline and selegiline (16.0% and 5.0%, respectively; Figure 5(D)). While safinamide use increased from Y2 to T0, rasagiline and selegiline prescription rates decreased. Among COMT inhibitors, opicapone was the most frequently used at T0 (Figure 5(E)); its use increased markedly from 1.0% of patients at Y2 to 8.0% at Y1 and 22.0% at T0. The use of entacapone decreased from 24.0% at Y2 to 20.0% at Y1 and 16.0% at T0. Tolcapone use remained stable, with <5% of patients using it at Y2, Y1, and T0.

At T0, 51.0% of patients (*n* = 150/296) had changed treatments, primarily due to fluctuations in treatment response (*n* = 219, 74.0%). Other reasons for treatment changes included tolerability problems (*n* = 47, 16.0%) and symptom persistence (*n* = 29, 10.0%). Across Y2 and Y1, fluctuations in treatment response were the leading cause of treatment change (∼80.0% of patients), followed by poor tolerability (∼5.0% of patients).

## 4. Discussion

The primary objective of the PD-FPA study was to describe how patients with long-standing, advanced PD and a fluctuating response to levodopa for ≥2 years were managed in clinical practice in Italy. This interim analysis includes results from the retrospective phase of the study, with real-world data on patient characteristics and treatment patterns at study entry and the 2 years prior to enrolment.

Despite patients having an extended disease duration, most were relatively stable over the 2-year retrospective analysis period, with scores indicating mild-to-moderate symptom severity at T0. The majority of patients had stage 2–3 PD, according to the Hoehn and Yahr Scale, indicating mild-to-moderate disease severity and physical independence. These findings were further supported by specific evaluations of motor/non-motor symptoms and motor complications, and by patient questionnaires.

With regard to the impact of PD on functioning and well-being, the PDQ-39 questionnaire highlighted a moderate disease impact, similar to that reported in other studies [21].

Non-motor symptoms were evaluated with NMSS and MDS-UPDRS. The mean total NMSS score of 59.1 in the current study was comparable with a pilot study (mean NMSS score of 56.5) with similarly aged PD patients who had shorter disease durations compared with the PD-FPA population (mean age 67.2 vs 68.0 years; mean disease duration 6.4 vs 12.6 years) [18].

According to the MDS-UPDRS (part IA and IB) assessment, important non-motor symptoms of PD, as judged by physicians, were anxiety and depression. However, important non-motor symptoms, as judged by patients, were fatigue, sleep problems, and daytime sleepiness. This finding suggests that some level of disagreement exists between physicians and patients in evaluating certain disease aspects, as has also been reported by other studies [24,25]. The evaluation of motor complications (MDS-UPDRS part IV) showed that, on average, patients with PD experienced dyskinesia for 23.0% of their total waking hours and spent 25.0% of their wake time in the OFF state. Interestingly, the time spent with dyskinesia and its functional impact on the patient was higher in females than in males, confirming previous findings that sex is an independent predictor of dyskinesia [26–28].

As expected, clinical assessments indicated wearing-off was the most common response fluctuation. The WOQ-19 questionnaire further highlighted that while most motor/non-motor symptoms improved with levodopa treatment, some of them (e.g. balance problems, difficulty in speech, anxiety, and mood changes) were more difficult to control, suggesting that mechanisms other than wearing-off might be involved.

Non-motor PD symptoms were particularly common in this study population, with anxiety and depression being amongst the most prevalent comorbidities and emerging as the most frequent non-motor symptom in response to wearing-off. Several studies have demonstrated that non-motor fluctuations are common and that patients often find these to be more disabling than motor symptoms [8,29–31].

In addition to describing the disease features of patients with advanced PD, the PD-FPA also aimed to document treatment patterns in this population, particularly the treatment of fluctuations. Across the 2 years prior to study entry, changes in therapy were frequent and primarily due to the emergence of fluctuations to treatment response. At study entry, safinamide and opicapone were the most frequently prescribed oral adjunctive therapies. Compared with the treatment patterns described in other observational studies of patients with advanced PD, the use of MAO-B inhibitors in PD-FPA was higher [32,33]. According to the international GLORIA registry, 35.0% of patients were using MAO-B inhibitors at study entry compared with 60.0% in the current study [32]. Data from the Italian population enrolled in the international OBSERVE trial also indicated that MAO-B inhibitors were used by only 23.0% of patients with advanced PD [33]. Similarly, a large, cross-sectional study from China reported that approximately 17% of patients who had a PD disease duration of >11 years were receiving selegiline [34]. COMT inhibitors were used by 41.0% of the PD-FPA population compared with 56.5% and 37.0% of the GLORIA registry and the OBSERVE study populations, respectively [32,33]. Notably, opicapone use increased markedly from 2 years prior to study entry, likely reflecting the fact that opicapone was granted marketing authorization in Italy in 2018 (i.e. Y1). The high proportion of patients that received levodopa with either COMT or MAO-B enzyme inhibitors may partially explain the relatively good disease control rate over 2 years prior to study entry. Analysis of the PD-FPA prospective data will provide further information on the use of these therapeutic strategies for managing fluctuations to treatment response.

Although the PD-FPA study population adequately describes patients within clinical practice in Italy, one limitation of our study is that the findings may not be generalizable to the global population with markedly different patient characteristics and treatment patterns. However, our study population had a remarkably long disease duration and, thus, might provide useful information on a PD subgroup that is underrepresented and poorly documented in the literature [35–37].

## 5. Conclusions

Managing patients with advanced PD and fluctuations in treatment response is challenging due to levodopa-associated complications, advanced patient age, comorbidities, and polypharmacy. The complex interplay between motor and non-motor symptoms also contributes to the heterogeneity of this condition. Individualized treatment pathways and specialized care are therefore warranted. This interim analysis of the Italian PD-FPA study suggests that levodopa-based oral therapy in combination with an adjunctive medication is a viable treatment option for patients with fluctuations in treatment response and an extended disease duration. Prospective data from the PD-FPA study will provide further information on the evolution and treatment of fluctuations to treatment response in clinical settings.

## Supplementary Material

Supplemental Material

## Data Availability

The data supporting the findings of this study are available from the study sponsor (Bial Italy) upon reasonable request (info.it@bial.com).
