# Supplementary material for: Advanced Parkinson’s disease treatment patterns in Italy: an observational study interim analysis
Source: Ann Med. 2024 Feb 21;56(1):2315226. doi: 10.1080/07853890.2024.2315226 (PMC10883087; doi:10.1080/07853890.2024.2315226)

**Advanced Parkinson’s disease treatment patterns in Italy: an observational study interim analysis**

**Supplementary Material**

**Authors:** Fabrizio Stocchi^1^, Paolo Barone^2^, Roberto Ceravolo^3^, Maria Francesca De Pandis^4-5^, Leonardo Lopiano^6^, Nicola Modugno^7^, Alessandro Padovani^8^, Manuela Pilleri^9^, Alessandro Tessitore^10^ and Mario Zappia^11^

**Affiliations:** ^1^Department of Neurology University San Raffaele Roma and IRCCS San Raffaele, 00166 Rome, Italy; ^2^University of Salerno, 84081 Baronissi, Salerno, Italy; ^3^ Neurodegenerative Disease Center, Department of Clinical and Experimental Medicine, University of Pisa, 56126 Pisa, Italy; ^4^ Department of Human Sciences and Promotion of Quality of Life, San Raffaele University, 00166 Roma, Italy; ^5^ San Raffaele Cassino Hospital, 03043 Cassino, Italy; ^6^ Department of Neuroscience Rita Levi-Montalcini, University of Turin; AOU Città della Salute e della Scienza, 10126 Turin, Italy; ^7^ I.R.C.C.S. Neuromed, 86077 Pozzilli, Isernia, Italy; ^8^ University of Brescia, 25100 Brescia, Italy; ^9^ UO Neurologia Casa di Cura Villa Margherita, 36057 Arcugnano Vicenza, Italy and Centro Parkinson e Parkinsonismi ASST Gaetano Pini CTO, 20122 Milano, Italy; ^10^ Department of Advanced Medical and Surgical Sciences, University of Campania "L. Vanvitelli", 80138 Naples, Italy; ^11^ Dept “G.F. Ingrassia”, University of Catania, 95123 Catania, Italy

**Corresponding author:** Fabrizio Stocchi

Email: fabrizio.stocchi@sanraffaele.it

**Supplementary Table S1.** Assessment of motor complications (MDS-UPDRS Part IV) in patients with advanced Parkinson’s disease and a fluctuating response to levodopa for ≥2 years.

|  | **Total (N = 294)^a^** | **Males (n = 176)** | **Females (n = 118)** |
| --- | --- | --- | --- |
| **A. Dyskinesias (exclusive of OFF-state dystonia)** | | | |
| Time spent with dyskinesias, n (%) | | | |
| Normal | 81 (27.5) | 61 (34.6) | 20 (17.0) |
| Slight–Mild | 175 (59.5) | 94 (53.4) | 81 (68.6) |
| Moderate–Severe | 38 (12.9) | 21 (11.9) | 17 (14.4) |
| % waking hours with dyskinesias | 23.0 | 21.0 | 26.0 |
| Functional impact of dyskinesias, n (%) | | | |
| Normal | 119 (40.5) | 83 (47.2) | 36 (30.5) |
| Slight–Mild | 139 (47.3) | 77 (43.8) | 62 (52.5) |
| Moderate–Severe | 36 (12.2) | 16 (9.1) | 20 (17.0) |
| **B. Motor fluctuations** | | | |
| Time spent in the OFF state, n (%) | | | |
| Normal | 8 (2.7) | 6 (3.4) | 2 (1.7) |
| Slight–Mild | 259 (88.1) | 154 (87.5) | 105 (88.9) |
| Moderate–Severe | 27 (9.2) | 16 (9.1) | 11 (9.3) |
| % of waking hours OFF | 25.0 | 27.0 | 23.0 |
| Functional impact of fluctuations, n (%) | | | |
| Normal | 22 (7.5) | 11 (6.3) | 11 (9.3) |
| Slight–Mild | 172 (58.1) | 104 (59.1) | 68 (57.6) |
| Moderate–Severe | 100 (33.8) | 61 (34.6) | 39 (33.1) |
| Complexity of motor fluctuations, n (%) | | | |
| Normal | 12 (4.1) | 8 (4.6) | 4 (3.4) |
| Slight–Mild | 240 (81.6) | 143 (81.3) | 97 (82.2) |
| Moderate–Severe | 42 (14.3) | 25 (14.2) | 17 (14.4) |
| **C. OFF dystonia** | | | |
| Painful OFF-state dystonia, n (%) | | | |
| Normal | 192 (65.3) | 116 (65.9) | 76 (64.4) |
| Slight–Mild | 84 (28.6) | 47 (26.7) | 37 (31.4) |
| Moderate–Severe | 18 (6.1) | 13 (7.4) | 5 (4.2) |
| % of OFF hours with dystonia | 12.0 | 13.0 | 12.0 |

MDS-UPDRS: Unified Parkinson’s Disease Rating Scale by the Movement Disorder Society.

^a^Only includes patients using oral levodopa therapy at study entry.

**Supplementary Table S2.** Reported use of dopaminergic drugs in the 2 years (Y2 and Y1) prior to study entry (T0).

| **Drug name, n (%)** | **Y2 (N=296)** | **Y1 (N=296)** | **T0 (N=296)** |
| --- | --- | --- | --- |
| Levodopa-carbidopa | 132 (44.6) | 134 (45.3) | 132 (44.6) |
| Levodopa-carbidopa MR | 64 (21.6) | 84 (28.4) | 98 (33.1) |
| Levodopa-carbidopa-entacapone | 60 (20.3) | 51 (17.2) | 42 (14.2) |
| Levodopa-benserazide | 92 (31.1) | 97 (32.8) | 99 (33.4) |
| Levodopa-benserazide CR | 2 (0.7) | 8 (2.7) | 8 (2.7) |
| Melevodopa-carbidopa | 86 (29.1) | 83 (28.0) | 109 (36.8) |
| LCIG | 3 (1.0) | 5 (1.7) | 9 (3.0) |

CR: controlled release; LCIG: levodopa-carbidopa intestinal gel; MR: modified release.

**Supplementary Table S3.** Reported use of dopamine agonists in the 2 years (Y2 and Y1) prior to study entry (T0).

| **Drug name, n (%)** | **Y2 (N=296)** | **Y1 (N=296)** | **T0 (N=296)** |
| --- | --- | --- | --- |
| Pergolide | 1 (0.3) | 0 | 1 (0.3) |
| Pramipexole | 74 (25.0) | 71 (24.0) | 58 (19.6) |
| Ropinirole | 57 (19.2) | 54 (18.3) | 50 (16.9) |
| Rotigotine | 64 (21.6) | 63 (21.3) | 56 (18.9) |
| Apomorphine | 4 (1.4) | 5 (1.7) | 5 (1.7) |
| Bromocriptine | 0 | 0 | 0 |
| Lisuride | 0 | 0 | 0 |
| Cabergoline | 0 | 0 | 0 |

**Supplementary Figure S1.** Reported comorbidities in the 2 years (Y2 and Y1) prior to study entry (T0).


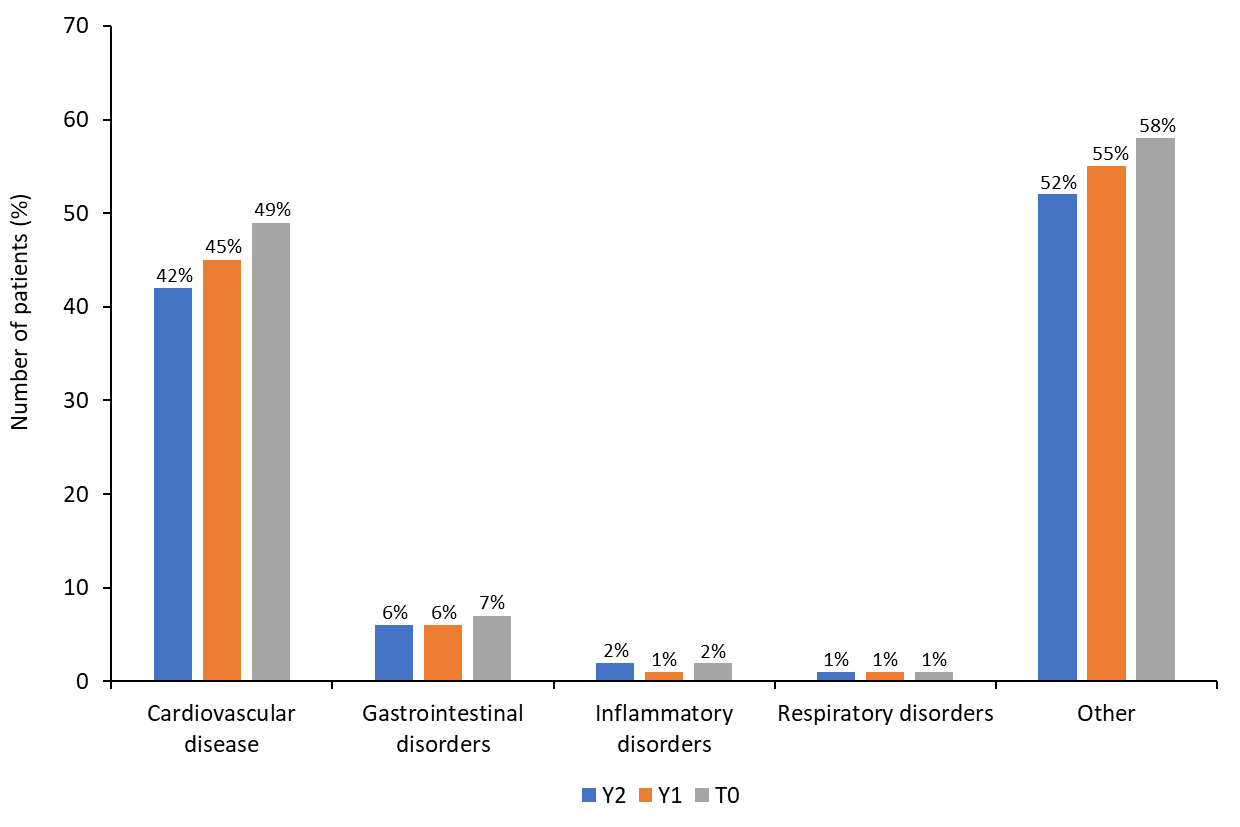

Supplement: Supplemental Material [file IANN_A_2315226_SM6235.docx]
